# The Heterogeneity of Consumer Preferences for Meat Safety Attributes in Traditional Markets

**DOI:** 10.3390/foods10030624

**Published:** 2021-03-16

**Authors:** Widya Satya Nugraha, Shang-Ho Yang, Kiyokazu Ujiie

**Affiliations:** 1 International Master Program of Agriculture, National Chung Hsing University, 145 Xingda Rd., South District, Taichung 40227, Taiwan; widyasatya@smail.nchu.edu.tw; 2 Graduate Institute of Bio-Industry Management, National Chung Hsing University, 145 Xingda Rd., South District, Taichung 40227, Taiwan; 3 Faculty of Life and Environmental Science, University of Tsukuba, 1-1-1 Tennodai Tsukuba-shi, Ibaraki 305-8572, Japan; ujiie.kiyokazu.gf@u.tsukuba.ac.jp

**Keywords:** heterogeneity, preferences, willingness to pay, WTP, market segmentation, food safety, attribute, labeling, traditional markets

## Abstract

In this study, we focus principally on Taiwan’s traditional markets, as food safety issues in those markets have been increasing recently. Thus, this poses pressures and challenges in traditional markets in terms of attracting consumers. This research aims to investigate whether there is consumer demand for more quality improvement from butchers and additional product information in Taiwan’s traditional markets by surveying consumers’ willingness to pay (WTP). This study determines consumers’ preferences for the important attributes and also investigates the different consumer segmentation in Taiwan’s traditional markets by analyzing the types of Taiwanese consumers who care about food safety and additional product information, including Taiwan Fresh Pork (TFP), QR code (provides product source information), Cold storage, and price. In this study, both Mixed Logit Model and Conditional Logit Model are used to elicit consumers’ WTP, and the Latent Class Model is used to understand the market segmentation in Taiwan’s traditional markets. The results show that the majority of Taiwanese consumers in traditional markets show preferences and WTP for meat products if Cold storage and QR code are available in Taiwan’s traditional markets. This work also provides appropriate strategies for improving the additional product information in Taiwan’s traditional markets, which can influence present and potential customers purchasing decisions.

## 1. Introduction

Food safety has become a topic of interest in the food industry, food policy, and academic research within the last 20 years. The consumers’ concern about food safety has been increasing during the past decades and has led governments globally to intensify their efforts to improve food safety [1]. Furthermore, consumers are becoming more aware of food safety issues as well as what they consume [2,3,4]. The unhealthy food choices will also cause health problems such as being linked to an increased risk of obesity, digestive problems, heart disease, stroke, cancer, and the most severe problem that occurs—early death [5,6]. Hence, food safety attributes serve as an indispensable role for consumers to determine which products to be purchased [7,8,9]. Consequently, food safety labeling and meat safety attributes have the potential to influence consumer purchase intentions in grocery stores because they could assist consumers in ensuring as well as guaranteeing food safety [10,11]. However, the investigations regarding meat safety attributes in traditional markets have not been widely observed [12,13,14]. An expanded in-depth examination of food safety labels and safety attributes, especially meat in traditional markets, needs to be explored further.

According to Helgi Analytics (2013), meat consumption in Taiwan continues to increase [15]. Furthermore, Taiwan is ranked within the top 5 countries with the highest pork consumption globally, making Taiwan one of the world’s largest pork consumption countries [16]. However, there has been a series of food safety scandals recently in Taiwan; for instance, Bovine Spongiform Encephalopathy (BSE), Salmonella, and Escherichia Coli (*E. coli*), have aroused tremendous attention among the public and media [17,18,19,20]. Taiwan’s pork industry has faced food safety challenges in various stages, especially in the food-safety chain [16]. In order to solve those issues, improving food safety has become a priority for the Taiwanese government.

In traditional markets, food safety attributes such as Cold storage (specific temperature storage of raw pork in order to maintain the freshness of the meat and increase the safety of the food), TFP (the label that emphasizes if the meat is domestically produced, fresh, hygenic, and safe), and QR code (containing information about product source information such as the source of the pig farm, the origin of the pork market, and the slaughter location and time) are normally not paid much attention by consumers [21,22]. However, as time goes by, food safety gains more attention from consumers locally and globally [21], but with limited understanding if there is a potential influence on consumer preferences. Hence, a study about consumers’ heterogeneity and identification of market segmentation are necessary to explore further, as well as the willingness to pay (WTP). For instance, when consumers in traditional markets want to buy meat products and the butchers provide food safety attributes with them, would the WTP as well as the purchasing decision be affected by extra food safety attributes? Therefore, it is interesting to further explore and examine this question.

Grunert (2005) and Losasso et al. (2012) have investigated that food safety issues also have an impact on consumers’ behavior and the mindset of the food business industry because price is no longer the most important factor [3,23]. Nonetheless, better-quality products have become the advantages in gaining market share and consumers’ trust. In addition, as in the global food markets, health is the most driving factor for trends and innovations [24], and the demand of food products providing food safety have increased [25]. Thus, many consumers have changed their buying behaviors and switched from shopping at traditional markets to more safer markets (modern markets or hypermarkets), as modern markets provided varieties of food safety-supporting facilities such as Cold storage and labeling as well as better service facilities [26]. However, it is still unknown whether consumers prefer labels as well as food safety attributes such as Cold storage and QR codes in Taiwan’s traditional markets, and whether Taiwanese consumers return to buy food products in Taiwan’s traditional markets or have different reactions.

Consequently, products with better food safety support facilities have a marketing advantage that producers can use to increase consumer consumption [27,28]. In addition, a great understanding of consumers’ needs is indispensable so that producers will react to consumers’ needs through generating safe foods [29,30]. Nevertheless, it should be noted whether the price of food products is expected to increase as a result of compliance with prerequisites for meeting specific standards, for instance, organic, Halal, and animal welfare. Different consumers tend to respond to the product attributes differently as they are heterogeneous in terms of socio-demographics (i.e., occupation and income), awareness, and food safety perceptions [31]. Thus, how to predict the heterogeneity of consumer preferences is interesting not only to the food industry but also to food policy makers and academic researchers [32].

As food safety has become a more crucial issue, many studies have investigated consumers WTP for food safety attributes in the Taiwanese context. For instance, these include food traceability system (FTS) [33], hypothetical safer seafood (HACCP) [34], country of origin labeling [35], animal welfare [36,37], hydroponically grown vegetables (HGV) to avoid the chemical residues [38], Halal meat label [39], and organic products [40]. However, those studies did not investigate consumers’ WTP for a specific type of meat safety attributes such as Cold storage, QR code, and meat labeling (i.e., the Taiwan Fresh Pork (TFP), also known as local fresh meat). In order to fill this gap, this research aimed to identify consumers’ WTP for those attributes as well as understand the different consumers’ segmentation and preferences in Taiwan’s traditional markets.

## 2. Materials and Methods

### 2.1. Empirical Model and Theoretical Framework

To assess consumer preferences for labeling effect and meat safety attributes, four attributes (TFP, QR code, Cold storage, and price) are used to create a discrete choice experiment (CE). The CE is based on the fundamental principles of economic theory [41] and the Random Utility Model (RUM) [42,43,44]. The underlying idea is that individuals are rational and will make their own decisions in order to maximize their utility based on their budget constraints. Afterwards, individuals shall select alternatives (from various options) in accordance with a function expressed as in Equation (1):

(1) $$U_{ij}=V_{ij}\left(Z_{j},N_{i}\right)+\varepsilon_{ij}$$

where $U_{ij}$ represents the utility from the alternative *j* for the respondent *i.* In this formula, also, there is a component of systematic utility $V_{ij}$, as well as the random component ($\varepsilon_{ij}$). $N_{i}$ represents the vector of socio-demographic respondents in the survey, and $Z_{j}$ represents the attribute in the scenarios inside the questionnaire. According to the formula in the CE model, individual *i* will constantly select alternative *j* in the choice experiments, which proposes that an individual *i* shall always choose alternative *j* that offers a higher utility. In terms of probability, if the consumer *i* will choose alternative *j*, then the model set-up can be shown in Equation (2):

(2) $$P_{ijt}=prob[(U_{ijt}>U_{ikt})\forall k\neq j]=prob[(V_{ijt}+\varepsilon_{ijt}>V_{ikt}+\varepsilon_{ikt})\forall k\neq j]$$

where the individual i would choose the alternative j under the situation t for labeling effect and meat safety attributes that provide a higher utility than the other alternative *k*. The $V_{ijt}$ represents a linear function of the four (4) meat safety attributes, including TFP, QR code, Cold storage, and price, while is described in Equation (3):

(3) $$U_{ijt}=V_{ijt}\left(Z_{i},N_{s}\right)+\varepsilon_{ijt}=\beta^{\prime }X_{ijt}+\varepsilon_{ijt}$$

where β represents the preference parameters which are consumer preferences, while $X_{ijt}$ represents the meat safety attributes in the alternative j under the situation t.

If we assume that consumer preference is homogeneous, this model implies a Conditional Logit Model (CLM), in which all β parameters for all attributes selected in the utility function are assumed to be constant across the sample. To reflect the heterogeneity of consumer preferences in the Mixed Logit Model (MLM) model, the observed variables normally depend on the parameter β; then, it will be distributed to all the respondents or consumers (shown in Equation (5)). The probability that the consumer i chooses the alternative j under situation t for labeling effect and meat safety attributes [45] can be expressed in Equation (4):

(4) $$P_{ijt}=\int \left\{\frac{exp\left(V_{ijt}\right)}{\sum_{k}exp\left(V_{ikt}\right)}\right\}g\left(\beta \right)d\beta$$

where g(β) expresses the probability density function of parameter β, and the distribution of β is determined by g(β) [25].

The Latent Class Model (LCM) is used to investigate consumers’ market segmentation for food safety supporting facilities in traditional markets. In the LCM, the consumer i selects alternative j in the situation *t* shown below in Equation (5):

(5) $$U_{ijt}{|}_{s}=\beta_{s}X_{ijt}+\varepsilon_{ijt}{|}_{s}$$

where $\beta_{s}$ represents the market segmentation, *s* is the parameter vector of the segment s associated with explanatory variables, and $\varepsilon_{ijt}{|}_{s}$ denotes the error term. Hence, in the LCM especially from *s* categories, the probability that the respondent *i* chooses option *j* can be performed in the formula in Equation (6):

(6) $$P_{ijt}=\overset{S}{\underset{s=1}{\sum }}G_{is}\;\overset{J}{\underset{j\;=\;1}{\prod }}P_{ijt}{|}_{s}$$

where $G_{is}$ is a consumer probability i included in the category s, $P_{ijt}{|}_{s}$ represents the probability that the consumer i chooses option j in the situation t from *s* categories [46]. Attribute levels of meat safety attributes (TFP, QR code, Cold storage, price) will have an effect exclusion of price. Moreover, the “Buyno” is a dummy variable, which has values of 0 or 1.

Based on the presumption that the price is a linear utility function, the average WTP of consumers for labeling effect and meat safety attributes can be calculated by the formula contained in Equation (7) [32,47,48]:

(7) $$WTP=-\frac{\beta_{j}}{\beta_{price}}.$$

The WTP for meat safety attributes in Taiwanese traditional markets will be calculated by dividing from the attribute coefficient ($\beta_{j}$) by the coefficient of the price ($\beta_{price}$).

### 2.2. Experiment Design and Choice Set

An interesting product in our study is pork, as Taiwan is one of the countries with the highest consumption of pork in the world [16]. Moreover, Taiwanese consumers are moderately concerned about food safety [34,49]. Thus, we also determine the alternative hypothesis (Ha) in this study, which is that Taiwanese consumers will have a positive response about the existence of food safety attributes in traditional markets. Hence, Taiwanese consumers may seek food safety before they make decisions. In this study, we include four (4) food attributes in the CE design, namely, TFP, QR code, Cold storage, and price.

This work studies consumer preferences for distinct attributes of meat safety based on a CE. The CE is based on the RUM [42] as well as Lancaster’s theory of consumer choice and its econometrics [41,50]. From the CE, we can evaluate the value of new products with new attributes [51]. Respondents need to make their choices among the options based on the principle of maximizing utility in the selection scenario [52] as the CE refers to a group of survey techniques to ask individual respondents about their preferences for estimating utility functions [53]. Moreover, product differences, as well as product utility, are decided through their attributes. A full-CE normally expects consumers to make several decisions in the multiple-choice scenario. Then, based on the consumer’s decision, their preferences could be evaluated and analyzed.

Theoretically, in this study, the full factorial design involves 4 × 2 × 2 × 2 = 32 choice sets. However, there are too many total alternatives for consumers to evaluate [46]. Thus, we followed the unrealistic choice set alternative [54] to reduce the number of choice sets. Since our research focuses on the traditional market, which is a very unique market venue [55,56], a pre-test from this study is also suggested to remove the unrealistic choice set alternative as well. Finally, all these unrealistic choice sets are removed, and four (4) choice sets of the CE scenarios are generated.

**Figure 1:**
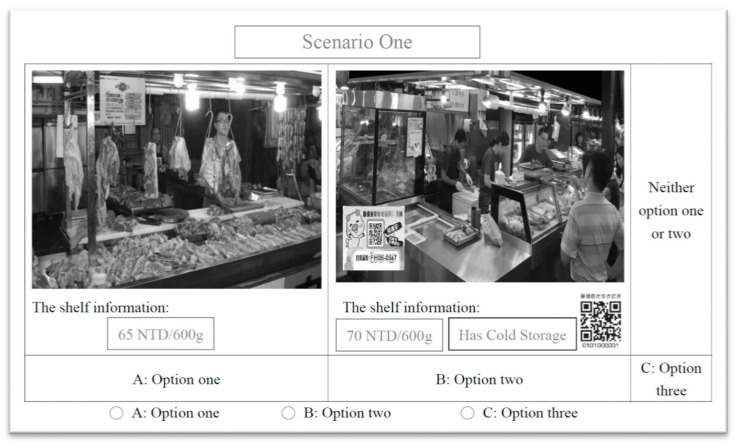
The example of a choice set.

In particular, respondents were asked to make a decision for different scenarios of buying a 600 g package of pork in traditional markets. In each choice set, they are asked to demonstrate their preference among two multi-attribute alternatives (options 1 and 2) and a “neither option 1 nor 2” option (3) [46]. Figure 1 displays a screenshot of a choice set.

### 2.3. Attribute and Level Settings

The appropriate attributes and levels are determined based on the actual sales situation in Taiwan’s traditional markets as well as market research that has been carried out in Taiwan’s traditional markets. Ultimately, a total of four (4) attributes, namely, TFP, QR code, Cold storage and price, were set to examine the heterogeneity consumer preferences for meat safety attributes in Taiwan’s traditional markets. Detailed information regarding the specific attributes as well as the level of each attribute is presented in Table 1.

In this study, QR code information is subdivided into two levels based on the supply chain process. Consumers can scan QR codes through their mobile devices to obtain product information such as pig farm location or slaughter time. A QR code not only enhances the trust of consumers but also strengthens the producer’s production responsibility. Meanwhile, the TFP brand emphasizes that the meat is “fresh, hygienic and safe”. All meat products are produced domestically and with high quality. Thus, consumers would be able to consume fresh and delicious pork products. The TFP attribute assumes one of two levels: providing the TFP label on pork products in Taiwan’s traditional markets and providing products without the TFP label.

The Cold storage attribute assumes one of two levels: provided Cold storage in Taiwan’s traditional markets and providing products without Cold storage. In order to improve the safety of meat, butchers use a temperature-controlled refrigerator to maintain the freshness of the meat, to isolate the dirty air in the traditional market, to control the temperature, and to prevent the growth of bacteria as well as to inhibit the growth of the number of bacteria in the meat. The weight specification of meat sold in traditional markets varies greatly depending on the buyers, resulting in a considerable distinction in price. To decrease the price distinction, the minimum weight specification of 600 g is utilized in the choice set. Furthermore, in order to avoid the amount of level effect [57], price attributes are set to four (4) levels based on the market research in Taiwan’s traditional markets: $65 NTD/600 g, $70 NTD/600 g, $75 NTD/600 g, and $80 NTD/600 g.

### 2.4. Questionnaire Design

In this study, we conducted an online survey through SurveyMonkey in May 2017. The survey aimed to sample respondents from all regions of Taiwan, including the South, Central, and North parts of Taiwan; thus, the results can be representative of the population. The questionnaire in this study includes three sections. The first section lists questions about the consumers’ habits related to the meat and food safety. In this section, we also apply screening questions to filter only respondents who actually buy meat in Taiwan’s traditional markets. The three screening questions follow: (1) in the past six months, have you visited any traditional market; (2) do you cook at home; and (3) are you the main grocery buyer in your family. Moreover, the second section presents a CE. In this section, each respondent is asked to answer four choice set situations. The last section is asking about the consumers’ socio-demographic variables, including age, gender, occupation, and religion.

## 3. Results

### 3.1. Sample Distribution

The descriptive statistics analysis in this study was used to depict Taiwan consumer preferences in terms of socio-demographics, purchasing behavior, and consumers’ habits. Table 1 describes the basic demographics of respondents as well as provides the details for all samples. Of the total number of respondents (904), more than half of the respondents are women (78%); this reflects that in Taiwan, females are responsible for purchasing in most households.

As observable from the mean value in Table 2, overall, we noticed that the average age of respondents is about 53 years old. The majority of the respondents have a higher education, which is above senior high school (68%). Moreover, most of the respondents are atheists (42%). Regarding the occupation category, about 25% of respondents work as housewives, 24% work in services, and 14% of respondents work in the manufacturing department. Furthermore, the vast majority of respondents usually visit traditional markets approximately 43.1 times in six months, and they visit supermarkets around 28.57 times in the same period. About 11% of the respondents said that their demand for meat or the meat consumption would increase in the future.

### 3.2. Heterogeneity in Consumer Preferences

This study aims to investigate the heterogeneity of Taiwanese preferences for meat safety attributes in traditional markets, including Cold storage, QR code, TFP, and price. In this study, we use two models, which are the MLM and Conditional Logit Model (CLM) to analyze Taiwanese preferences’ heterogeneity. In order to know which model is more appropriate, we can examine through the score of the information criteria or the goodness of fit from the model such as Log-Likelihood, Akaike Information Criterion (AIC), and Bayesian Information Criterion (BIC). The higher value of Log-Likelihood information criteria and the lower value of AIC and BIC information criteria, the more suitable the model. In short, all measures that are log-likelihood, AIC, and BIC imply that the MLM demonstrates a better fitting model than the CLM.

Moreover, according Table 3, the standard deviation of the MLM is statistically significant, indicating that the respondents do have heterogeneous preferences; thus, this will make the MLM a more appropriate model [58]. Additionally, the derived standard deviation parameters for meat safety attributes, including TFP, QR code, Cold storage, and price, are significantly different from zero. It implies that there is heterogeneity in the population in terms of respondents’ preferences for meat safety attributes in Taiwan’s traditional markets. Moreover, the Cold storage attribute also has a higher standard deviation from the estimated parameter compared to the other attributes. On the other hand, there is a high heterogeneity among surveyed consumers for meat safety attributes.

The estimation of the MLM is reported in Table 3. The MLM results indicate that the coefficient of “buy no” is negative and statistically significant, which means that the respondents could get a higher utility from choosing any alternative to the “neither” option. Furthermore, respondents are also willing to purchase the product that provides meat safety attributes including TFP, QR code, and Cold storage. As expected, the coefficient for the price is negative and statistically significant, indicating that the price increments decrease consumers’ utility as well as lower the probability to buy, which is also consistent with previous studies by [59,60,61].

According to the coefficient estimation for the meat safety attributes, the descending order of relative importance is Cold storage (2.668), TFP (1.171), and QR code (1.066). Therefore, except for price and “buy no”, Cold storage is the most important attribute for the consumers. All food safety attributes have a significant coefficient, although the level of the coefficients is different. Thus, this finding is very interesting because even though there are no food safety attributes in traditional markets such as Cold storage, TFP, and QR code, Taiwanese consumers are still interested in those attributes.

Additionally, it can be seen that Cold storage have the highest coefficient (2.668) among food safety attributes. The coefficient of the Cold storage attribute is positive and statistically significant with the *p*-value < 0.01, suggesting that Taiwanese consumers prefer Taiwan’s traditional markets with Cold storage offers. This finding is the consequence of the increasing consumer awareness of food safety in Taiwan’s traditional markets; thus, the consumers want to improve the food safety attributes such as providing Cold storage in traditional markets. Furthermore, Taiwanese consumers think that Cold storage is a crucial attribute in traditional markets to reduce food safety problems. Next, TFP and QR code also have a positive significance (1.171 and 1.066, respectively) with the *p*-value < 0.1, indicating that Taiwanese consumers are willing to accept as well as prefer pork products in Taiwan’s traditional markets with a TFP label and QR code. These findings may imply that Taiwanese consumers in traditional markets may prefer the benefits of food safety attributes, including Cold storage, TFP, and QR code.

Table 3 also presents the interaction terms between the main attributes (TFP, QR code, and Cold storage) and socio-demographic variables in the MLM. In Table 3, interaction terms between the demographics, including Age, Gender, Education, Atheist, Manufacture, Housekeeper, Traditional Market, Supermarket, and Demand to buy meat, were introduced to measure the impact of basic demographics on food safety attributes choices. Among the Taiwanese consumers, the interaction terms with age that have a significantly negative coefficient are TFP × Age (−0.018) and Cold storage × Age (−0.012), indicating that younger consumers in Taiwan have a stronger TFP label as well as Cold storage preferences. Subsequently, Atheist X TFP and Atheist X Cold storage have a positive and significant coefficient (0.581 and 0.439), exhibiting that non-religious people in Taiwan are more likely to prefer Taiwan’s traditional markets, which provide cold storage and the TFP label. In contrast, QR code X Atheist shows a negative and significant coefficient (−0.324), indicating that Taiwanese consumers who identify with a religion prefer meat products in traditional markets with QR codes, as consumers could access the information related to the meat products.

Moreover, both occupation categories TFP × Service (0.573) and TFP × Manufacture (0.736) have a positive and significant coefficient, suggesting that Taiwanese consumers working in the manufacturing and service sectors are more likely to prefer pork with a TFP label in Taiwan’s traditional markets. Furthermore, Cold storage × Traditional Market has a negative and significant coefficient (−0.004), which implies that consumers who frequently visit traditional markets do not prefer the existence of cold storage in traditional markets. It may imply that consumers who frequently go to traditional markets may have their usual purchasing preference; thus, it may create a negative perception when cold storage is provided. Next, Cold storage × Demand buy meat (0.526) has a positive and significant coefficient, indicating that if the demand for meat product increases in the future, consumers are more likely to prefer cold storage availability in Taiwan’s traditional markets.

Finally, in the study, we hypothesized (H_0_) that Taiwanese consumers shall negatively respond to the existence of food safety attributes in traditional markets; however, this study’s findings also support our hypothesis or reject H_0_. According to the results, we can confirm that Taiwanese consumers prefer and accept food safety attributes (Cold storage, TFP, and QR code) in traditional markets. Thus, this work suggests that the Taiwanese government could reduce food safety problems in traditional markets by implementing those food safety attributes. Therefore, it will potentially generate benefits for consumers and will be able to attract consumers to buy meat products in traditional markets.

### 3.3. The Standard Deviation of the Random Parameter

Table 4 shows the results of the variances of the random parameters. In this result, we try to specify the coefficients to be independently distributed, and one would expect correlation. For instance, the effect of one attribute could be positive or negatively correlated with the other attributes (Buy no × Cold storage). However, we can assume that the random parameters are correlated. In this step, researchers usually check the correlation between variables before estimating the standard deviation in the MLM. According to the coefficient estimated for the results, almost all variables tend to have a high positive correlation and significant covariance with other attributes. Nevertheless, for the Cold storage attribute with Buy no, TFP and QR code have a negative and significant covariance. The correlation between Buy no × Buy no is significantly positive, showing that there is a higher correlation (6.283) compared with other variables. Thus, it indicates that Buy no × Buy no shows the strength of a strong linear relationship, explaining why consumers do not want to buy meat in traditional markets: there are no food safety attributes in traditional markets. For the Cold storage variable correlation, all have a negative significance with other variables, including TFP, QR code, and Buy no. For instance, a negative correlation from Buy no × Cold storage (−0.925) indicates that if traditional markets do not provide cold storage for storing meat and keeping the meat fresh, consumers do not want to buy meat in traditional markets.

### 3.4. The WTP

Taking the MLM as a representation of the distribution of heterogeneity in consumer preferences for food safety attributes in Taiwan’s traditional markets, we could discuss the impact that consumers’ food safety risk concerns have on their WTP. Table 5 summarizes the dependent variables’ definitions and descriptive statistics as well as individual-specific mean willingness to pay (MWTP) for each food safety attribute and explanatory variable: the respondents’ socio-demographics. It shows that the WTP estimates for each food safety attribute differ across choice model; thus, it indicates that scenario selection is important and tends to have a significant on the implications of parameter estimates. The standard deviation of the WTP estimates are relatively high (i.e., the variation in coefficient is fairly substantial), indicating that consumers tend to respond quite differently and are considerably heterogeneous in preferences as well as valuations for meat safety attributes including TFP, QR code, Cold storage, and price.

Results from the MLM suggest that on average, consumers are willing to pay $29.169 NTD/600 g more for meat products with a QR code and $73.006 NTD/600 g more for Cold storage. In conclusion, if we try to provide Cold storage and QR code in traditional markets, the total of WTP is $102 NTD/600 g. In Table 1, the four (4) price levels are set according to the base price of 80 NTD/600 g, and we can roughly interpret that consumers are willing to pay a premium of 22 NTD/600 g for pork products with a QR code as well as if traditional markets offer Cold storage, as shown. This study also shows that the mean WTP for Cold storage is higher than QR code, as demonstrated by higher estimates of the standard deviation. The result shows that there is a strong need for the Taiwanese government to provide adequate food safety in traditional markets.

Table 5 also shows the WTP for food safety attributes among socio-demographics in Taiwan’s traditional markets, respectively. Although Taiwanese consumers are generally concerned about food safety, they are heterogeneous in their WTP a price premium to cover the cost of providing safety attributes, which varies considerably. The WTP for cold storage and TFP is higher for Taiwanese consumers who have no religion and believe that their demand for pork consumption will increase in the future. On the other hand, those respondents will likely be more willing to pay a higher price to obtain better food safety attributes. The MWTP results derived in this study indicate that consumers are willing to pay a positive amount for food safety attributes (Cold storage and QR code). This may give the government and food industry sector confidence and an incentive to invest in quality improvement for food safety in traditional markets. Conclusively, higher quality pork in terms of having additional safety attributes can be sold at higher prices in Taiwan’s traditional markets.

### 3.5. The Estimated Results of the LCM

The LCM analyzes the heterogeneity of market segmentations in Taiwan’s traditional markets from the perspective of differences in group preferences. However, a key issue in the LCM is defining the number of consumer segments. In order to select the optimal number of segments in the latent classes, we could use the minimum Akaike Information Criterion (AIC) and the minimum Bayesian Information Criterion (BIC) [62].

**Figure 2:**
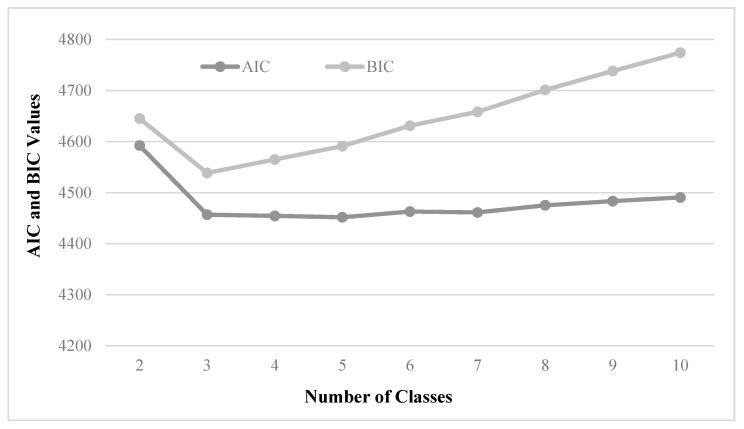
The information criteria (AIC and BIC) used in determining number of the classes in the Latent Class Model (LCM). AIC: Akaike Information Criterion, BIC: Bayesian Information Criterion.

Figure 2 presents the statistical summary about the information criteria, including AIC and BIC, for classes 2 to 10. For the AIC, the suggested optimal number of the latent class is five. However, the suggested optimal number for the BIC is three classes. Moreover, the marginal changes in AIC for four and five classes are considerably smaller than those in three classes, indicating that adding segments to five classes does not result in much improvement. Furthermore, when we tried to run the data for classes above three, we encountered a few problems: some estimated values of the coefficient were not provided and the Log-likelihood was not concave, or the convergence was not achieved. Consequently, three classes provide a parsimonious description of the latent class structure. In conclusion, according to the values of AIC and BIC criteria, the LCM with three (3) classes is the best model to be estimated.

As shown in Table 6, in the LCM for consumers in segment 1, the part-worth utilities of QR code and Cold storage levels are positive and significant at *p*-value < 0.01, and this result corresponds to the results in the estimation of mean WTP that used in the MLM. Respondents in the first class are the majority class; we named this class as “Food Safety Conscious” (70% of the respondents) as they care more about food safety issues, especially in traditional markets. Thus, they have the strongest preference for meat safety attributes, which are Cold storage and QR code. However, in class 1, the TFP does not show significance, indicating that the standard for obtaining food safety in Taiwan’s traditional markets is relatively low, causing an impression on Taiwanese consumers that pork with the TFP label may not much different from the one with no labeling. The LCM results in class 1 confirm that the majority of the consumers in Taiwan’s traditional markets are indeed aware of food safety.

The LCM in the class 2 represents the “QR code Fans” consumers as well as the minority market segmentation in Taiwan’s traditional markets (6.1% of the respondents), indicating that they are those preferring to buy pork products with a QR code in Taiwan’s traditional markets. This may also reveal that the strategy of adopting a QR code would receive at least 6% of consumers’ attention. The latent class 3 shows a relatively high price coefficient value (in absolute value terms) relative to the other attributes’ coefficients, indicating that this market segmentation is price sensitive. Since the consumers in class 3 are very interested in paying economical prices for the pork products, thus, we call this class “Price Conscious”.

Following the increasing of food safety awareness in traditional markets, this work has further confirmed that the majority of consumers in traditional markets in Taiwan prefer meat products provided with food safety attributes such as Cold storage and QR code. Furthermore, the results of this study also reject the null hypothesis, which states that consumers usually would not care about the availability of food safety attributes in traditional markets. Some news reports mentioned that Taiwanese prefer (Taiwanese prefer warm-body pork: https://english.cw.com.tw/article/article.action?id=2803, accessed on 27 October 2020) “warm-body pork”, which is never chilled or frozen; the consumers who do not prefer the Cold storage attribute in the latent class 3 seem to be an indication of the warm-body pork market share (i.e., 23.4%). If the butchers are reluctant to adopt cold storage in traditional markets because of the demand of warm-body pork, it may be an evidence that the consumers’ preference in the small market share (the latent class 3) dominates over the majority (the latent class 1) of consumers’ preference.

## 4. Conclusions

Taiwanese consumers are paying increasing attention to food safety issues; thus, it is important to understand their preferences, the WTP, as well as market segmentation. This study investigates the heterogeneity of consumer preferences for meat safety attributes in traditional markets with the four attributes, including TFP, QR code, Cold storage, and price. This study is based on the CE conducted among 904 Taiwanese respondents. The MLM was estimated to elicit consumers’ WTP for the four attributes, and the LCM was applied to understand the market segmentation in traditional markets in Taiwan. The main conclusions are as follows.

The results from both the MLM and the LCM indicate that although surveyed consumers are in the majority concerned about food safety, they are heterogeneous in their WTP for a price premium to cover the cost of providing safety attributes. The MLM suggests that Cold storage is considered as the most preferable attribute, which is followed by TFP labels, and then the last favored is QR codes. For the Cold storage attribute, we found that consumers prefer the availability of cold storage in traditional markets and are willing to pay an additional price for this attribute, because it can maintain the meat’s freshness and improve meat safety. Furthermore, Taiwanese consumers more prefer pork in traditional markets with TFP labels and QR codes. Moreover, consumers are willing to pay an additional price for those attributes because TFP labels can guarantee that the meat is fresh, hygienic, and safe. Furthermore, QR codes could increase the consumers’ trust, as consumers can check the information on the source of pork products.

It was expected that different demographic groups would react differently to the availability of food safety attributes in Taiwanese traditional markets. The results of the interaction terms from the MLM explain that people who are more interested in food safety attributes (TFP, QR code, Cold storage) tend to have a higher demand for meat as well as work in the manufacturing and service sectors. Moreover, the younger generation and consumers who do not identify with a religion reach more positively and are more concerned about food safety.

The results of the LCM indicated that consumers in Taiwan have heterogeneous preferences for meat safety attributes. Based on the consumers’ varying preferences for meat safety attributes, they can be delineated into three classes: “Class 1 (Food Safety Conscious), Class 2 (QR code Fans), and Class 3 (Price Conscious)”. Among the three classes of consumers, “Class 1 (Food Safety Conscious)” is the major group in Taiwan’s traditional markets (70.5% market share). They are more concerned about the food safety; thus, in this group, they are looking for any indication to assure that the product is safe, and they are less focused on price. Since the consumers in this group are very concerned about food safety, there is a positive significance for Cold storage and QR code attributes. It indicates that the majority of Taiwanese consumers prefer Taiwan’s traditional markets to provide cold storage and QR codes. “Class 2 (QR code Fans)” is the minority group (6.1%), and in this group, the consumers only care about QR codes, or we can say the consumers strongly prefer QR codes. “Class 3 (Price Conscious)” is the middle group (23.4%), and the consumers care most about price and would pay limited attention to the food safety attributes.

Although it is commonly known that in traditional markets, consumers usually do not pay much attention to food safety attributes, including Cold storage, QR code, and TFP. It is surprising to find out that the majority of the consumers (70.5%) in Taiwan’s traditional markets indeed are food safety conscious and consider attributes such as Cold storage and QR codes. Overall, Taiwanese consumers are willing to pay $22 NTD/600 g higher when Cold storage and QR code attributes are given. Nevertheless, a potentially small number of consumers may be negatively affected due to the personal preference for food safety attributes. Yet, the majority of consumers in Taiwan are more interested by those benefits of the food safety attributes, especially cold storage and QR codes. In conclusion, in order to attract more consumers in Taiwan’s traditional markets, the government and food industry sectors may focus on those attributes.

This study’s findings can provide guidance to policy makers and the food industry to develop appropriate marketing strategies and provide different types of food safety attributes for different groups of consumers in Taiwan’s traditional markets. This finding also provides managerial implication for the government as well as the food industry. To enhance consumers’ demand for these products and convey more benefit to the consumers, the government and the food industry may amplify the supervision of pork products’ quality and safety. At the same time, they may focus on cold storage, followed by QR code and TFP. Meat product sellers and butchers in traditional markets can improve their profits by targeting one more consumer segment. For instance, they could provide cold storage in traditional markets as well as put QR codes on the pork products; thus, they can attract more consumers who are more aware of food safety, whose market share is the largest and WTP for food safety attributes is the highest. On the other hand, the Taiwanese government may strengthen public education and promote food safety scientific publications, especially in traditional markets.

As mentioned in the previous section, this work examined only Taiwanese consumers’ preferences; hence, the limitation in this study is the lack of understanding the preference of the government and the stakeholders other than consumers. Due to this limitation, meat product sellers and butchers cannot obtain information of other stakeholders’ preferences from this work. Moreover, studies employing surveys of other stakeholders could establish valuable research in the future.

## Figures and Tables

**Table 1:** Attributes and levels used in the CE design.

Attributes	Levels
TFP	Provide TFP label in Taiwan’s traditional markets
	No TFP
QR code	Provide QR code in Taiwan’s traditional markets
	No QR code
Cold storage	Provide Cold storage in Taiwan’s traditional markets
	No Cold storage
Price	65 NTD/600 g
	70 NTD/600 g
	75 NTD/600 g
	80 NTD/600 g

Source: From this research.

**Table 2:** Definitions and sample statistics of variables (*n* = 904).

Variables	Descriptions	Mean	Standard Deviations
Female	DV = 1 if the respondent is female	0.78	0.41
Age	CV; the respondent’s age	53.14	9.33
Education	DV = 1 if the respondent has education above senior high school	0.68	0.46
Atheist	DV = 1 if the respondent does not identify with a religion	0.42	0.49
Manufacture	DV = 1 if the respondent’s job is in manufacturing	0.14	0.35
Service	DV = 1 if the respondent’s job is in service	0.24	0.43
Housewife	DV = 1 if the respondent’s job is housewife	0.25	0.43
Traditional market	CV; the frequency of the respondent going to traditional markets	43.10	34.53
Supermarket	CV; the frequency of the respondent going to supermarkets	28.57	28.34
Demand buy meat	DV = 1 if the demand of the respondent’s buying meat will increase in the future	0.11	0.59

Source: From this research. Note: DV means the dummy variable; CV means the continuous variable.

**Table 3:** The estimation of the Mixed Logit Model (MLM) and Conditional Logit Model (CLM).

Attributes	CLM		MLM			
	Coef. Estimation		Coef. Estimation		Standard Deviation	
Price	−0.038	***	−0.037	**		
Buy no	−3.829	***	−4.670	***	2.507	***
TFP	0.999	**	1.171	*	0.774	***
QR code	0.456		1.066	*	0.992	***
Cold storage	1.732	***	2.668	***	1.700	***
**Interaction Term**						
TFP × Age	−0.017	**	−0.018	*		
TFP × Female	0.202		0.127			
TFP × Education	−0.150		−0.241			
TFP × Atheist	0.420	***	0.581	***		
TFP × Service	0.294		0.573	**		
TFP × Manufacture	0.611	**	0.736	**		
TFP × Housekeeper	−0.021		−0.075			
TFP × Traditional Market	−0.001		0.000			
TFP × Supermarket	−0.001		−0.002			
TFP × Demand buy meat	−0.195		−0.344			
Cold storage × Age	−0.009	*	−0.012	**		
Cold storage × Female	0.045		−0.022			
Cold storage × Education	−0.140		−0.181			
Cold storage × Atheist	0.265	***	0.439	***		
Cold storage × Service	−0.083		−0.084			
Cold storage × Manufacture	0.135		0.141			
Cold storage × Housekeeper	−0.122		−0.202			
Cold storage × Traditional Market	−0.003	*	−0.004	**		
Cold storage × Supermarket	0.001		0.001			
Cold storage × Demand buy meat	0.324	**	0.526	***		
QR code × Age	0.002		0.001			
QR code × Female	−0.120		−0.149			
QR code × Education	0.038		0.039			
QR code × Atheist	−0.297	**	−0.324	*		
QR code × Service	−0.059		−0.167			
QR code × Manufacture	−0.068		−0.084			
QR code × Housekeeper	0.020		0.005			
QR code × Traditional Market	−0.001		−0.003			
QR code × Supermarket	0.001		0.002			
QR code × Demand buy meat	0.244		0.515	*		
Log Likelihood:	−2432.8		−2187.2			
AIC:	4935.627		4464.315			
BIC:	5152.386		4743.005			
Number of observations:	10,848					

Source: From this research. Note: *** *p* < 0.01, ** *p* < 0.05, * *p* < 0.1.

**Table 4:** Estimation for the random parameters.

Attributes	Coef. Estimate		Std. Error
Buy no × Buy no	6.283	***	1.368
Buy no × Cold storage	−0.925	**	0.446
Buy no × QR code	1.449	***	0.487
Buy no × TFP	1.477	***	0.430
Cold storage × Cold storage	0.599	*	0.357
Cold storage × QR code	−0.527	*	0.273
Cold storage × TFP	−0.497	***	0.191
QR code × QR code	0.984	***	0.335
QR code × TFP	1.548	***	0.301
TFP × TFP	2.890	***	0.410

Source: From this research. Note: *** *p* < 0.01, ** *p* < 0.05, * *p* < 0.1.

**Table 5:** Estimation of the mean willingness to pay (MWTP) using the MLM.

Main Effect	Mean (NTD/600 g)		Standard Deviation	Standard Error
Buy no	−127.800	***	67.757	25.327
TFP	32.039		20.919	25.236
QR code	29.169	*	26.811	17.308
Cold storage	73.006	**	45.946	33.821
**Interaction Term**				
TFP × Age	−0.491		-	0.351
TFP × Female	3.485		-	6.454
TFP × Education	−6.600		-	7.503
TFP × Atheist	15.910	*	-	8.810
TFP × Service	15.677		-	9.588
TFP × Manufacture	20.141		-	12.635
TFP × Housekeeper	−2.048		-	6.880
TFP × Traditional Market	−0.002		-	0.080
TFP × Supermarket	−0.058		-	0.098
TFP X Demand buy meat	−9.400		-	9.358
Cold storage × Age	−0.327		-	0.217
Cold storage × Female	−0.589		-	3.761
Cold storage × Education	−4.960		-	4.575
Cold storage × Atheist	12.003	**	-	6.026
Cold storage × Service	−2.302		-	4.247
Cold storage × Manufacture	3.847		-	5.349
Cold storage × Housekeeper	−5.539		-	4.802
Cold storage × Traditional Market	−0.103		-	0.066
Cold storage × Supermarket	0.022		-	0.058
Cold storage × Demand buy meat	14.380	*	-	8.035
QR code × Age	0.020		-	0.236
QR code × Female	−4.064		-	5.721
QR code × Education	1.064		-	5.518
QR code × Atheist	−8.875		-	6.130
QR code × Service	−4.568		-	6.233
QR code × Manufacture	−2.294		-	7.261
QR code × Housekeeper	0.127		-	5.841
QR code × Traditional Market	−0.089		-	0.078
QR code × Supermarket	0.054		-	0.084
QR code × Demand buy meat	14.100		-	9.649

Source: From this research. Note: *** *p* < 0.01, ** *p* < 0.05, * *p* < 0.1.

**Table 6:** Estimated results of the LCM.

Variable	Estimates		
	Class 1 “Food Safety Conscious”	Class 2 “QR Code Fans”	Class 3 “Price Conscious”
Price	−0.019	−0.034	−0.088 **
Buy No	−2.179	−0.864	−9.439 ***
TFP	0.365	−0.043	0.275
QR code	0.880 ***	0.813 **	0.104
Cold storage	2.259 ***	0.479	−0.443 **
Probability Class	0.705	0.061	0.234
/Share1	1116 ***		
/Share2	−1335 ***		
Log Likelihood:	−2211.353		
AIC:	4456.706		
BIC:	4555.422		
Number of Obs:	10,848		

Source: From this research. Note: *** *p* < 0.01, ** *p* < 0.05.

## Data Availability

Data of the current study are available from the corresponding author on reasonable request.
